# Water depth influences survival and predator‐specific patterns of nest loss in three secretive marsh bird species

**DOI:** 10.1002/ece3.10823

**Published:** 2023-12-11

**Authors:** Stephanie M. Schmidt, Auriel M. V. Fournier, Joshua M. Osborn, Thomas J. Benson

**Affiliations:** ^1^ Illinois Natural History Survey, Prairie Research Institute University of Illinois at Urbana‐Champaign Havana Illinois USA; ^2^ Stephen A. Forbes Biological Station, Illinois Natural History Survey, Prairie Research Institute University of Illinois at Urbana‐Champaign Havana Illinois USA; ^3^ Department of Natural Resources and Environmental Sciences University of Illinois at Urbana‐Champaign Havana Illinois USA

**Keywords:** marsh bird, nest predation, nest survival, water depth, wetland management

## Abstract

Wetlands have become increasingly rare in the United States, negatively influencing wetland‐dependent birds, and many remaining wetlands are intensively managed through seasonal dewatering mimicking historic flood pulses during spring and summer. However, water around nests may provide protection from terrestrial predators, and lowering water levels during the breeding season of wetland birds may increase predation risk and exacerbate marsh bird population declines. Understanding interactions between water depth, nesting marsh birds, and nest predators is critical to aid managers in developing a multi‐species management approach in emergent wetlands. During the 2020 and 2021 breeding seasons, we examined nest survival of 148 marsh bird nests (American Coot, *Fulica americana*, *n* = 1; Common Gallinule, *Gallinula galeata*, *n* = 64; and Least Bittern; *Ixobrychus exilis*, *n* = 83) and installed cameras at 78 nests to identify predators at a large, restored floodplain wetland in Illinois where the primary management technique is seasonal water removal to stimulate germination of moist soil plants. We found nest predation of, and abandonment by, Least Bittern and Common Gallinule were related to shallower water, and early season, high volume dewatering. Least Bitterns nested more commonly along wetland edges and nests farther from the shore were more likely to survive. Similarly, we found mammalian depredation of nests and nest abandonment decreased when deeper water was present around nests. Alternatively, snake predation was observed earlier in the year prior to water removal from inundated emergent vegetation. Our results demonstrate water depth may be an important deterrent of nest predators, especially mammals, during the breeding season. Further, we recommend managers delay dewatering until after the nesting season at sites where management for conservation‐priority marsh birds is a focus.

## Graphical abstract

The timing and intensity of water removal for wetland management may have adverse effects on nesting marsh birds because water can be important for limiting predator access to nests. We examined the impacts of water depth on survival and predation risk at Least Bittern, Common Gallinule, and American Coot nests, and we found deeper water below and around nests was critically important for facilitating nest survival, especially when faced with raccoon and mink predators. Our study suggests water removal should occur outside of peak nesting stages and at a lower intensity at wetlands where marsh bird nest survival is a concern.

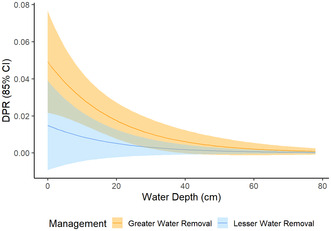

## 1 INTRODUCTION

The United States has experienced substantial wetland loss, primarily due to drainage for agriculture or urban development (Dahl, 1990; Holland et al., 1995). By the 1980s, around 53% of the United States' colonial‐era wetlands had been drained, mostly driven by Midwestern states where most remaining wetlands are small and isolated (Dahl, 1990; Havera et al., 1997). These landscape changes have led to altered hydrology, as well as changes in interspersion and size of emergent vegetation communities within remaining wetlands (Havera et al., 1997). Floodplain wetlands, which historically relied on overbank flooding from the river to set back succession, have faced additional challenges as humans have channelized and added levees to rivers, resulting in increasing river flow rate, changes in flooding seasonality, and a hydrologic disconnection from the adjacent floodplain (Pierce & King, 2013).

Wetland loss and degradation have significantly contributed to the decline of wetland wildlife, including marsh birds (Eddleman et al., 1988; Newman et al., 2003; Quesnelle et al., 2013; Rosenberg et al., 2019; Soulliere et al., 2018; Zedler & Kercher, 2005). Secretive marsh birds (i.e., rails, bitterns, night‐herons, grebes; hereafter marsh birds) are cryptic species that are associated with emergent wetlands for all aspects of their life cycle (Bradshaw et al., 2020; Darrah & Krementz, 2010; Harms & Dinsmore, 2013; Wilson et al., 2018). To reverse the negative effects of marsh loss and protect at‐risk species, wetland managers have actively restored and managed emergent wetlands to benefit a variety of wetland birds (waterfowl, marsh birds, shorebirds), and research suggests that wetlands restored by removing agricultural fields from production quickly provide habitat for breeding birds of conservation concern (Fairbairn & Dinsmore, 2001; Fournier et al., 2021; Rundle & Fredrickson, 1981; Vanausdall & Dinsmore, 2019; vanRees‐Siewert & Dinsmore, 1996).

Managed wetlands often have infrastructure for managers to artificially manipulate water levels to mimic a wetland's natural dynamic hydrology using pumps, weirs, and subsurface drainage structures (Guhin & Hayes, 2015). Managers use periodic dewatering, or the active removal of water, to create moist‐soil plant communities that feed migrating waterfowl (i.e. *Polygonum* spp., *Panicum* spp.; Bellrose, 1941, Fredrickson & Taylor, 1982), and enhance natural wetland functions such as improving water quality, providing flood protection, recharging groundwater, and controlling nuisance species such as non‐native fish and frogs (Guhin & Hayes, 2015). Additionally, seasonally managed water levels and periods of flooding are imperative for controlling nuisance vegetation, such as willow, cottonwood, cocklebur, and ash (Bellrose, 1941; Lane & Jensen, 1999). Water‐level management can also be used to create favorable habitat conditions for priority taxa, including spawning fish (Guhin & Hayes, 2015; Lemke et al., 2017), mammals (Lane & Jensen, 1999; Weller & Spatcher, 1965), invertebrates (Lemke et al., 2018), shorebirds (Lemke et al., 2018; Ma et al., 2010), grassland birds (Finch, 2016; Żmihorski et al., 2016), and marsh birds (Fournier et al., 2019). While active dewatering is widely used and the benefits for waterfowl and other priority taxa have been well documented, the impacts of active dewatering on nesting marsh birds remain understudied (Malone et al., 2023).

Although dewatering can be beneficial to a variety of wetland‐dependent species, the timing and volume of water removal may impact the suitability of conditions for breeding marsh birds and may expose nests to a more diverse predator community (Lowther, 1977). Whereas marsh bird nests already experience relatively high failure rates and nest predation is likely the primary cause, removing water from nesting habitat during the breeding season has the potential to negatively impact marsh bird populations and increase predation risk by altering habitat structure and removing a natural barrier to depredation (i.e., surrounding water; Ricklefs, 1969, Ma et al., 2010, Fournier et al., 2021). Previous studies of nesting birds have found influences of visual and olfactory cues such as nest concealment and nest activity, distance from shore, and water depth on predation risk, the impacts of which may be influenced by a predator's identity (Báldi & Batáry, 2005; Batáry & Báldi, 2004; Colombelli‐Négrel & Kleindorfer, 2005; Jedlikowski et al., 2015; Martin et al., 2000; Post, 1998; Skutch, 1949). Specifically, nest concealment has been linked to risk from aerial predators and increased water depth and distance from edge and large unfragmented wetland areas has been associated with decreased risk from land‐based predators (Báldi & Batáry, 2005; Batáry & Báldi, 2004; Ellis et al., 2020; Hoover, 2005; Picman et al., 1993). For instance, Frederick and Collopy (1989) hypothesized that as little as 5–10 cm of water can substantially restrict the movements of mammalian predators. However, nest predation is not well‐explained solely by broad habitat generalizations but may also be an artifact of predator identity, behavior, and activity (Benson et al., 2010; Lyons et al., 2015). Generally, predator identity and their relationship with habitat conditions that exacerbate predation risk are largely unknown and research has yet to elucidate this relationship because camera studies have not been used extensively in wetlands (DeGregorio et al., 2016). We evaluated the impact of water depth and water level management on nest survival of marsh bird nests. To better understand the influence of wetland management on conservation priority species, we examined links between water depth and predator‐specific patterns of nest predation of three obligate marsh birds, Least Bitterns (*Ixobrychus exilis*), Common Gallinules (*Gallinula galeata*), and American Coots (*Fulica americana*). Two of these species, Least Bittern and Common Gallinule, are recognized as species of conservation concern across the U.S. (IESPB, 2020; Soulliere et al., 2018). We expected increased predation associated with shallower water, particularly by mammals, and at nests with stronger visual and olfactory cues to alert predators, such as nests with larger clutches.

## 2 METHODS

### 2.1 Study area

**FIGURE 1:**
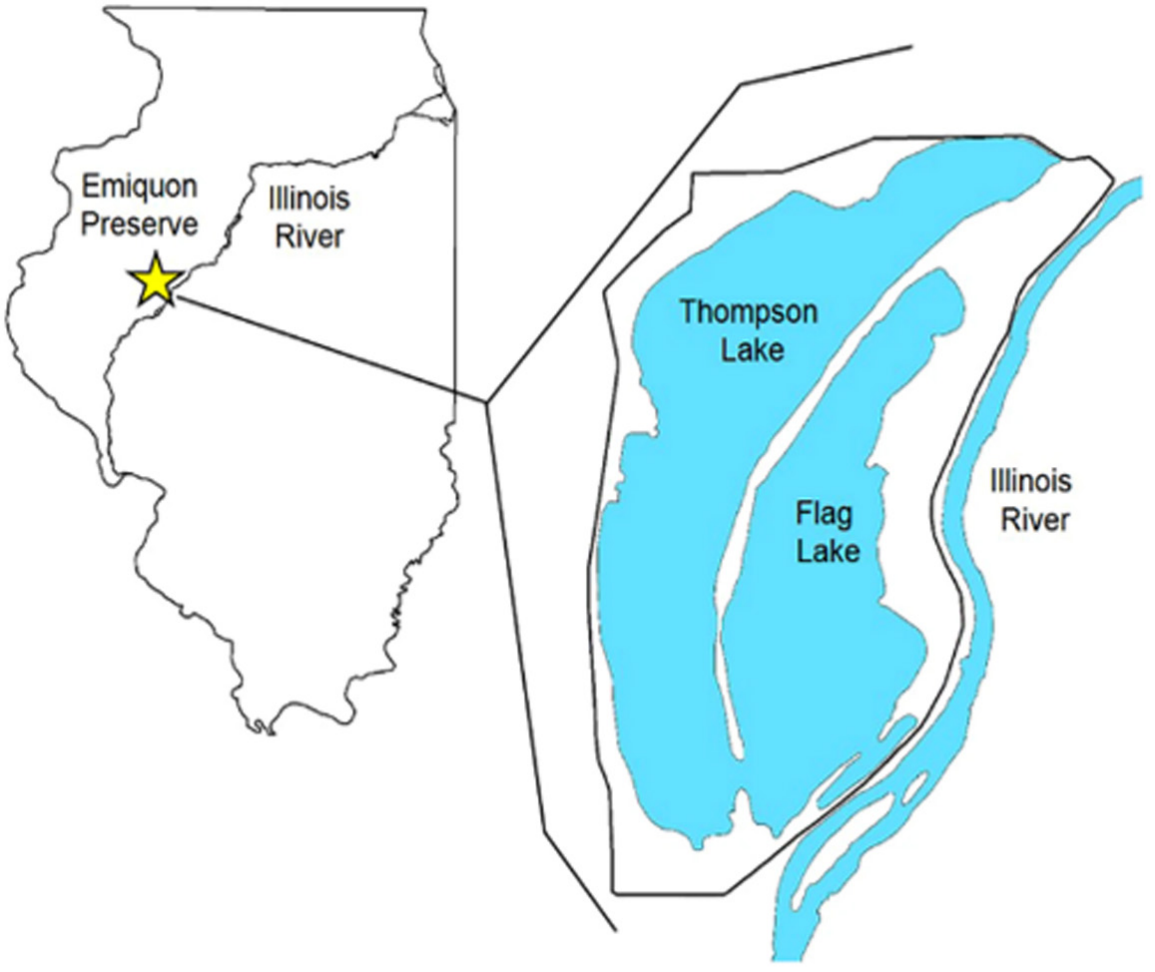
Emiquon Preserve is located west of the LaGrange Reach (river miles 121–126) of the Illinois River in Fulton County, Illinois, USA. Emiquon Preserve consists of Thompson Lake, Flag Lake, and surrounding vegetation (Hine et al., 2017).

Emiquon Preserve in Fulton County, Illinois is a 2723‐ha floodplain Ramsar Wetland of International Importance managed by The Nature Conservancy and is one of the largest floodplain wetland restorations in the Midwest (Chen et al., 2017). Few wetland restoration projects are as heavily monitored through key ecological attributes to inform future restoration efforts like Emiquon Preserve has been, lending to its high quality and distinction in the region (Lemke et al., 2018). Emiquon Preserve consists of two lakes, Thompson Lake and Flag Lake (Figure 1), and these backwater lakes once supported the most productive floodplain ecosystem in Illinois before they were disconnected from the Illinois River in the early 1900s and a levee was constructed (Chen & Lemke, 2009; vanMiddlesworth et al., 2015). Today, The Nature Conservancy uses two electrically powered 32,000 gpm pumps to practice cyclical dewatering with years of intense dewatering followed by years of moderate or minimal dewatering. This approach halts marsh succession to the lake‐marsh phase, mimics a natural flood pulse that would occur prior to disconnection from the Illinois River, and increases habitat heterogeneity to benefit a variety of waterbird species (Ma et al., 2010; van der Valk & Davis, 1978).

### 2.2 Study species

In the breeding seasons of 2020 and 2021, we selected American Coot, Common Gallinule, and Least Bittern as our focal species because of their dependence on emergent wetlands, diversity of nest structures, and confirmed nesting at Emiquon Preserve in past research (Fournier et al., 2021). The American Coot is a native diving rail that nests in secured floating platforms on reedbed edges inundated by ≤76 cm of water (Bent, 1963; Lane & Jensen, 1999). The Least Bittern is a threatened species in Illinois and a regional species of conservation concern (IESPB, 2020, Soulliere et al., 2018). This small wading bird builds suspended nests in dense growths of vegetation ≤1.2 m above 7–96 cm of water and typically within 10 m of open water (Bent, 1963; Weller & Spatcher, 1965). The Common Gallinule is morphologically similar to the coot and exhibits similar nest site selection; however, the Common Gallinule is less common and is endangered in Illinois (Fredrickson, 1971; IESPB, 2020; Weller & Spatcher, 1965).

### 2.3 Survey site selection and nest searching

In 2020, we randomly selected 81 nest‐searching plots in persistent emergent vegetation (*Typha* spp.), hemi‐marsh (50:50 interspersed water‐cover ratio; Weller & Spatcher, 1965), and boundaries between the two vegetation communities. We targeted these vegetation communities using ArcGIS data generated from a concurrent study delineating the wetland communities at the Emiquon Preserve (Osborn et al., 2021; ESRI, Redlands, California). The vegetation cover map was not available at the time of site selection during spring 2021, so nest‐searching plots were randomly selected from current persistent emergent vegetation and hemi‐marsh conditions at Emiquon Preserve in May 2021. We created plots using randomly selected distances (25–75 m) and directions from the vegetation edge at selected points. The randomly selected point in both years defined the center of a survey plot.

Nest‐searching plots were 50‐m by 50‐m squares, and we searched for nests by systematically traversing these plots in teams of 1–3 in transects ≤2 m apart to minimize the risk of missing nests. We selected this plot size because it was large enough to provide ample opportunities for locating nests while still manageable for our teams to search given the dense vegetation and the concealment of the marsh bird nests. Although it is possible, we failed to find some nests using this approach, and it was found to be effective in prior research (Fournier et al., 2021). We systematically searched plots from 05:00–13:00 (CDT) from 12 May through 31 July, 2020, and 16 May through 17 July, 2021. Each plot was searched 1–3 times throughout the season with a 2–3‐week interval between searches at the same plot. We stopped visiting a plot if no nests were found after two visits or focal species were absent. We also performed incidental searches for nests outside of defined plots in areas with ideal plant communities and conspicuous activity of our focal species. Due to the nature of the dense emergent vegetation at survey plots, we minimized trampling vegetation by traveling on foot through naturally open spaces outside of our survey plot to not link open edges with nests in the dense interior and refraining from re‐breaking vegetation along survey paths during nest rechecks.

We recorded GPS coordinates at all nests and marked with flagging tape within the vicinity to facilitate relocation. Nests located along survey routes were flagged 5‐meters from the nest along the survey route at eye‐level or greater, and nests located incidentally were flagged in a similar fashion in two opposing cardinal directions. Alternatively, nests that received a nest camera were flagged at the camera box, located 5‐meters or more from the nest. All flags were placed in the direction biologists would travel to reach the nests and were encountered first.

We aged eggs using a field candler and photographs of incubation stages for representative eggs adjusted to fit the incubation length for our focal species (Hanson, 1954; Hanson & Kossack, 1957; Weller, 1961; Young, 1988). We categorized nest age based on the oldest egg. If the nest contained nestlings when first found, we did not assign an age. Nests were revisited every 3 or 5 days weather permitting (range of 1–6 days) to determine fate and collect habitat measurements, and nest visits were approximately 2–10 minutes in length dependent on the presence or absence of a camera. We stopped visiting when nests were terminated (abandoned or depredated) or fledglings departed the nest. A nest was determined to be abandoned if there was no evidence of an active incubator (i.e. eggs cold, eggs not aging, and incubator absent on camera) and determined to be depredated if all eggs or hatchlings were missing before reaching hatching or fledging age or a predator was seen on camera removing nest contents.

### 2.4 Video cameras

We identified predators and examined predator‐specific responses to habitat variables using miniature video cameras at a subset of marsh bird nests selected for their varied habitat conditions and anticipated hatch date. Nest cameras were disguised using paint and concealed by natural vegetation to minimize any impact they may have (Chiavacci et al., 2018; Cox et al., 2012; Herranz et al., 2002). The cameras were small, measuring approximately 3 cm by 4 cm, and used infrared light‐emitting diodes (LEDs) to facilitate recording in low‐light conditions. We used black paint to camouflage cameras before mounting them on a wooden or metal dowel ≥15 cm from nests with an unobstructed view of the nest contents from above. We suspended the cable from the camera above water where it led to a waterproof box ≥5 m from the nest. The box was camouflaged with black paint and placed out of view of the nest in dense vegetation. The box also housed a digital video recorder (DVR), which recorded continuously at a rate of 8 frames per sec, and a 20‐Amp‐hour sealed lead acid battery (Cox et al., 2012). Unlike past studies of nest predators in terrestrial systems (DeGregorio et al., 2016), it was necessary to develop a system that could adapt to changing water levels. Consequently, we mounted each waterproof box on a 0.6 by 0.9 m piece of 5 cm thick foam insulation anchored in place with a wooden or metal dowel to keep it from floating away. We exchanged SD cards and batteries every 3 days weather permitting (range 1–4 days) adjusting camera angles if nest contents were out of frame, until nests failed or young departed from the nest. We reviewed videos to determine hatch and depredation dates and to identify predators; nests were classified as abandoned if an incubating adult did not return within 24 hours, and cameras remained at nests until the nest failed or nestlings were unreliably on the nest.

### 2.5 Nest context and temporal/biological measurements

We aimed to determine how nest context and temporal/biological variables influenced overall predation risk as well as risk from specific classes of predators. We recorded nest‐site variables during nest checks, including nest height (cm), water depth under the nest (cm), average height of emergent vegetation (cm) between the tallest and shortest piece of vegetation within 1 m of the nest, clutch size, nest stage (incubating or hatched), day of year, and nest fate. In an effort to minimize disturbance at nests during active incubation or trample vegetation, we did not take habitat measurements further than 1 m from the nest.

Water‐level management in 2020 was more intense than in 2021, with 1.37 m of water removed in 59 days in 2020 compared to 0.46 m of water removed in 26 days in 2021. In 2021, we recorded four additional variables that we thought might be related to predation risk at nests. We measured vegetation density by recording the average number of stems within a 30 × 30 cm square adjacent to the nest at a randomly selected cardinal direction and two equidistant points 0.5 m from the nest. At nest visits in 2021, we also estimated visibility above a nest (%), and percent cover by emergent vegetation to determine habitat openness (open, hemi‐marsh, dense) and dominant vegetation type (persistent emergent, nonpersistent emergent, floating leaved) within a 2.5 m radius of the nest We defined hemi‐marsh as 40% to 60% vegetation to 60% to 40% water. We determined distance of each nest to a continuous shoreline using ArcGIS (ESRI, Redlands, California), daily water height above sea level recorded at Emiquon Preserve, and a 15 cm contour map of the preserve for each day a nest was visited.

### 2.6 Statistical analyses

To examine the effects of cameras on predation risk, we compared survival rates at nests with and without cameras following the same three‐day visit schedule to reduce bias associated with human presence at the nest. We investigated the effects of visit frequency using only nests without cameras with longer and shorter intervals between nest visits. For both analyses, we used a logistic exposure method with the *glm* function in R and we present our results as coefficient estimates (*β*) and 85% confidence intervals (R Core Team, 2013; Shaffer, 2004). We used 85% rather than 95% confidence intervals for consistency with our AIC results (details below).

To examine nest survival, we were interested in the potential influence of both habitat‐related factors that may be influenced by management actions, such as water depth, vegetation structure, and distance from shore (hereafter nest context variables) as well as temporal variables, such as day of year, and variables such as clutch size and nest stage that, although not directly influenced by management actions, have been demonstrated to influence nest survival in other species (hereafter temporal/biological variables) (Knutson et al., 2007). We were primarily interested in the influence of nest‐context variables and therefore fit candidate models containing these time‐varying covariates in a first stage, ranking these models using an information‐theoretic approach corrected for small sample sizes (AIC_c_; Burnham & Anderson, 2002). We then carried forward competitive models from this first stage (defined as ΔAIC_c_ < 2) to a second stage where we examined whether these competitive nest‐context models fit better (using AIC_c_) than temporal/biological variables often found to influence survival in prior studies. Because we were interested in predictors of nests surviving to produce young, we grouped all causes of egg and chick loss together in models, including abandonment as a form of failure in cases where an incubator was confirmed (i.e., was not abandoned when originally found), and considered nests as surviving each interval if they had eggs or chicks and remained actively attended by an adult. We analyzed nest survival for each species separately using logistic exposure models (R Core Team, 2013, Shaffer, 2004).

Our nest‐context models included effects of water depth, distance to shore, nest height, stem density, nest visibility, habitat openness visually estimated within 2.5 m of the nest, average vegetation height, and year of greater/lesser water removal. Our candidate‐model set included a constant‐survival model, single‐variable models, additive models that included two‐ and three‐variable combinations of our variables of primary interest, water depth, distance to shore, and year of greater/lesser water removal, and an interactive model that combined nest height with vegetation height (Table S1). Our temporal/biological candidate models included single‐variable models with nest stage, clutch size, and day of the year (i.e., ordinal day) in addition to a constant‐survival model. We did not include highly correlated variables (|*r*| > .7) in the same model, and model sets were the same across species except for omitting multiple‐variable models for Common Gallinule that included distance to shore given the lack of variation for this species relative to Least Bitterns (Table S2).

We assessed predator‐specific nest losses as a function of nest context and temporal/biological variables using data from video‐monitored nests of all three nesting species using multinomial regression. As above, we were interested in the influence of nest context and temporal/biological variables on daily predation risk by mammals, snakes, birds, and other sources of failure (abandonment and unknown predators), and we divided our variables into five categories (mammal, snake, bird, other, and survive). We included all complete predation events, and for nests that were visited multiple times by a predator, we included only the first partial predation event as including all events would overestimate predation risk (Báldi & Batáry, 2005; Lyons et al., 2015). We ran models with the *multinom* function in the *nnet* package in R using daily intervals from our videos and held predictor values constant between visits, and we evaluated support for these models using AIC_c_ (Burnham & Anderson, 2002; R Core Team, 2013). We considered models within eight ∆AIC_c_ units of the top model to be competitive, as opposed to two ∆AIC_c_ units because of additional parameters in a multinomial analysis (i.e., each added variable results in four additional parameters, a different coefficient for each failure group). As above, we ranked the nest‐context models and temporal/biological models against competitive nest‐context models, and we based inferences on coefficient estimates and their corresponding 85% confidence intervals with variables considered meaningful when confidence intervals did not overlap zero. We chose 85% rather than 95% confidence intervals because the conclusions regarding support for variables (i.e., based on coefficient intervals not overlapping zero) tend to be more congruent with model selection based on AIC (Arnold, 2010).

## 3 RESULTS

### 3.1 Dewatering timing and volume

**FIGURE 2:**
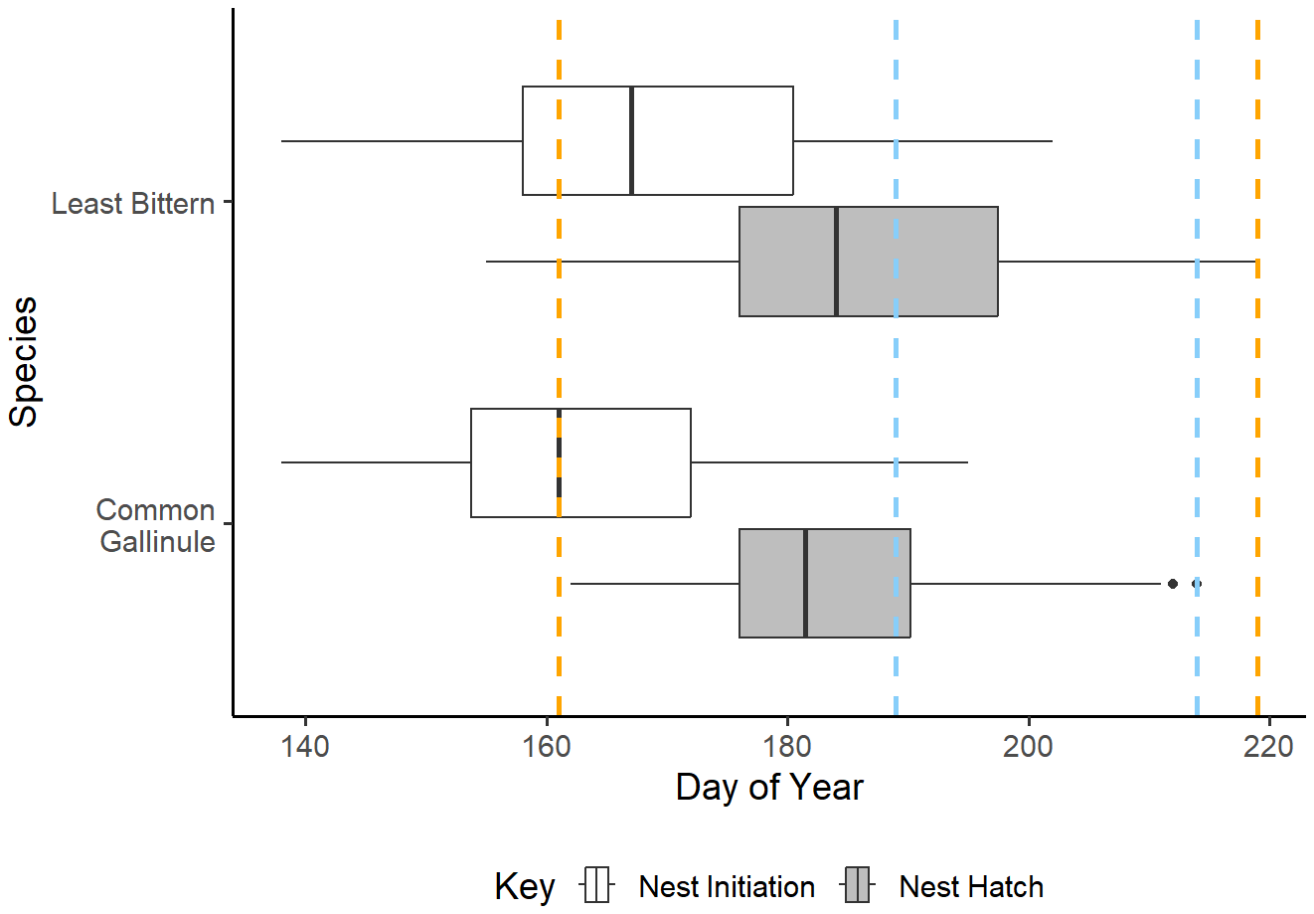
Nest initiation dates and hatch dates, of Least Bitterns (*Ixobrychus exilis*) and Common Gallinules (*Gallinula galeata*) in 2020 and 2021 as well as the time period water was actively draw off the landscape, shown as start and end dates in 2020 (orange) and 2021 (blue), at Emiquon Preserve, Illinois, USA. Day 140 corresponds to May 19. Peak nesting and hatching dates, defined based on the first and third quartiles, were similar across years.

Water depth under nests averaged 36.4 cm (±19.49 cm [SD]) and ranged from 0 to 78 cm in 2020 and 0 to 84 cm in 2021. Distance from shore averaged 329.29 m (±331.08 m [SD]) and ranged from 0 to 1161.76 m in 2020 and 0–765.90 m in 2021. In 2020, active dewatering was initiated on June 9 and lasted through August 6 and resulted in a net loss of ~1.37 m of water, and in 2021, active dewatering occurred between July 8 and August 2 and resulted in a net loss of ~0.46 m of water. There was substantial overlap between peak nesting dates, defined as quartiles 1–3, and active dewatering timing in 2020 but not in 2021 (Figure 2). Additionally, distance to shoreline for the duration of incubation changed on average − 139.29 m (±197.20 m [SD]; range: −793.37 m–522.01 m) in 2020 and −42.38 m (±113.80 m [SD]; range: −456.91 m–276 m) in 2021.

### 3.2 Nesting phenology and predators

#### 3.2.1 Nesting phenology

We monitored 148 nests (*n* = 1 American Coot, *n* = 83 Least Bittern, *n* = 64 Common Gallinule) in 2020 and 2021 (69 in 2020, 79 in 2021). In 2020 and 2021, nest initiation and expected hatch dates revealed Least Bitterns nested between June 3 and July 20 and Common Gallinules between June 1 and July 17. Multinomial analyses of nest predation included the American Coot, while nest survival analyses focused only on Least Bitterns and Common Gallinules.

#### 3.2.2 Predators

We used cameras to monitor 78 nests in 2020 (*n* = 43) and 2021 (*n* = 35), and we observed 38 events leading to nest loss in 2020 (*n* = 26) and 2021 (*n* = 12). The most common predation events were committed by mammals and was comprised of raccoons (*Procoyon lotor*) (*n* = 5), American mink (*Neovision vision*) (*n* = 3), and unidentified mammals (*n* = 2), which were a result of cameras only capturing fur or glowing eyes during a predation event. The second most common group were snakes (*n* = 5), all Eastern Fox Snakes (*Pantherophis vulpinus*), and birds (*n* = 4), consisting of Common Gallinules, Marsh Wrens (*Cistothorus palustris*), and Brown‐headed Cowbirds (*Molothrus ater*). The final group defined as other, encompassed unknown losses (*n* = 10) and abandonments (*n* = 9). Unknown losses were recorded when a camera failed to identify the cause of nest loss either because cameras or nests were knocked down or cameras failed due to battery failure or damaged camera equipment. For that reason, it is unclear if nests were depredated before or after abandonment, hence their inclusion in a category together. Raccoon predation resulted in complete nest loss four out of five times and the one partial predation event occurred at a nest where juveniles were force‐fledged. The majority of mink and fox snake events were partial predation events and often involved the same species, presumably the same individual, returning to the nest later to consume the remaining eggs or nestlings. All avian events involved the individual breaking rather than consuming a few eggs. Successful nests that incurred a partial predation event were not common, and partially depredated nests generally failed later.

### 3.3 Nest survival and predation

#### 3.3.1 Predator cameras and visit frequency

Cameras remained at nests for 11.9 days on average (10.9 days in 2020, 13.1 days in 2021), and we found no effect of visit frequency on nest survival for Least Bitterns (*β*_visit frequency‐LEBI_ = −0.86; 85% CI: −1.79 to 0.07) and Common Gallinules (*β*_visit frequency‐COGA_ = −0.54; 85% CI: −1.37 to 0.29) or cameras on nest survival for Least Bitterns (*β*_cameras‐LEBI_ = 0.21; 85% CI: −0.09 to 0.51; Table 1). Four Least Bittern nests were abandoned (three with cameras, one without). Cameras were associated with Common Gallinule nest survival with six observed abandonments at camera monitored nests (*β*_cameras‐COGA_ = −0.74; 85% CI: −1.37 to −0.11; Table 1).

**TABLE 1:** Parameter estimates for the effects of nest cameras and visit frequency on marsh bird nest fate at the Emiquon Preserve during 2020 and 2021.

Marsh bird species	Disturbance	Coefficient (*β*)	85% Confidence interval
Least Bittern	Camera	0.2094	−0.0921 to 0.5109
	Visit Frequency	−0.8561	−1.7835 to 0.0713
Common Gallinule	Camera	−0.7375	−1.3656 to −0.1094
	Visit Frequency	−0.5403	−1.3739 to 0.2933

#### 3.3.2 Species‐specific nest survival

**FIGURE 3:**
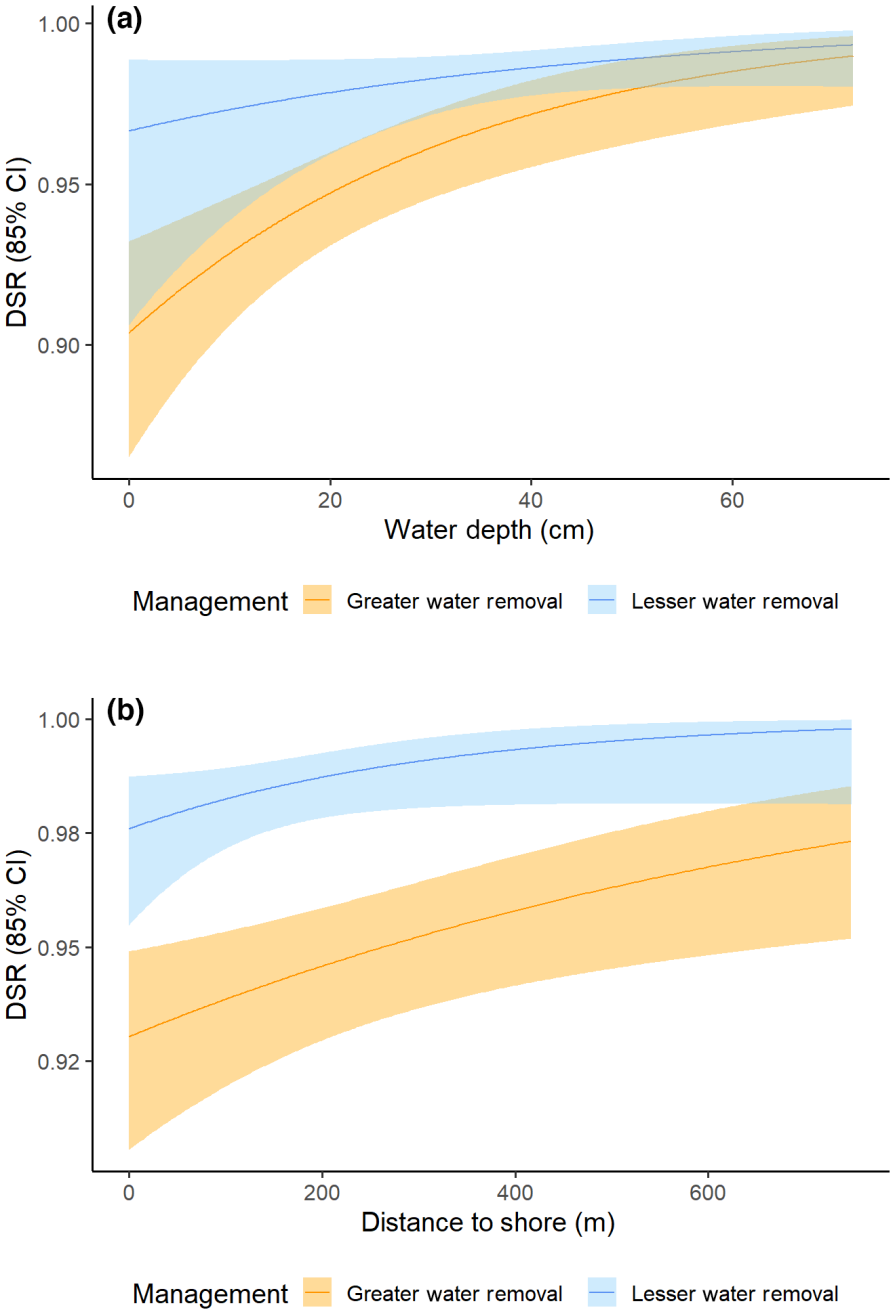
Daily survival rate (DSR) of Least Bittern nests (*n* = 83) in a year of high water management intensity (2020) and a year of low water management intensity (2021) at Emiquon Preserve, Illinois, USA, as a function of water depth (cm) and management intensity/year (a) and distance to shore (m) and management intensity/year (b).

**FIGURE 4:**
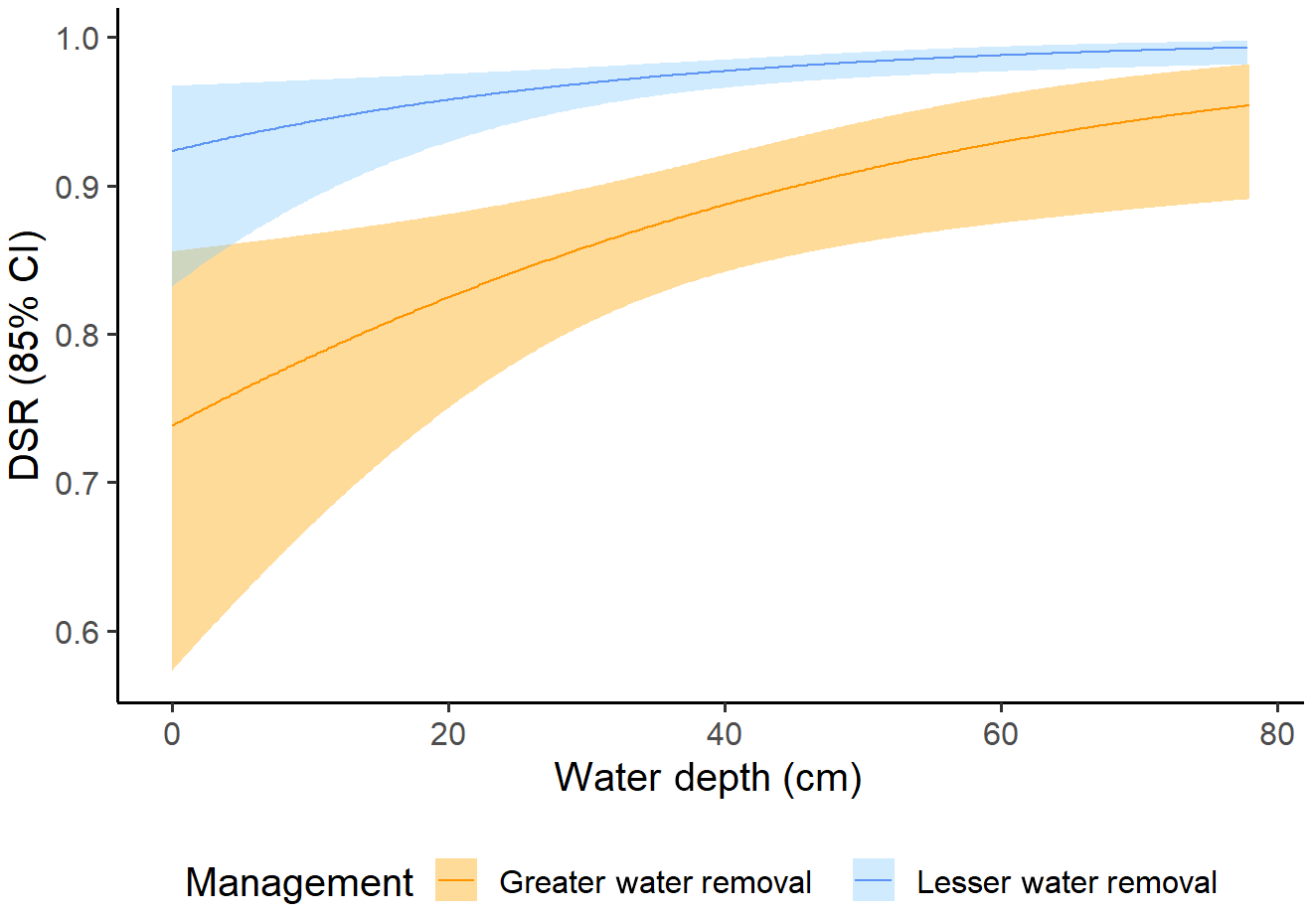
Daily survival rate (DSR) of Common Gallinule nests (*n* = 64) in a year of high water management intensity (2020) and a year of low water management intensity (2021) at Emiquon Preserve, Illinois, USA, as a function of water depth (cm) and management intensity/year.

Our best‐fit model for survival of Least Bittern nests (*n* = 83) contained water depth, distance to shore, and year of greater/lesser water removal (*w*_*i*_ = 0.84). There were no other competitive nest context or temporal/biological models. Daily survival rates were lower for nests over shallower water (*β*_water depth 2020_ = 0.0324; 85% CI: 0.0164 to 0.0484; *β*_water depth 2021_ = 0.0225; 85% CI: −0.0046 to 0.0496), closer to shore (*β*_distance to shore 2020_ = 0.0013; 85% CI: 0.0003 to 0.0023; *β*_distance to shore 2021_ = 0.0032; 85% CI: −0.0003 to 0.0067), and in the year of greater water removal (2020) compared to in the year of less water removal (2021) (Figure 3). For Common Gallinules (*n* = 64), the top model contained water depth and year of greater/lesser water removal with no other competitive models (*w*_*i*_ = 0.80). Common Gallinule nest survival also was lower over shallow water and in the year of greater water removal (2020) (*β*_water depth_ = 0.0256; 85% CI: 0.0063 to 0.0449) relative to the year of less water removal (2021) (*β*_water depth_ = 0.0317; 85% CI: 0.0101 to 0.0533) (Figure 4). Daily survival rates for Least Bittern and Common Gallinule nests increased from 0.9704 (CI: 0.9463–0.9721) to 0.9897 (CI: 0.9818–0.9942) and from 0.9376 (CI: 0.9050–0.9600) to 0.9825 (CI: 0.9721–0.9891) between 2020 and 2021, respectively (Table 2).

**TABLE 2:** Least Bittern and Common Gallinule daily survival rate and survival to hatch using year‐only models at Emiquon Preserve, Illinois, USA in 2020 and 2021 with 85% confidence intervals.

Species	2020		2021	
	DSR^a^	Survival to hatch^b^	DSR	Survival to hatch
Least Bittern	0.9704 (0.9463–0.0.9721)	0.60 (0.39–0.62)	0.9897 (0.9818–0.9942)	0.84 (0.73–0.91)
Common Gallinule	0.9376 (0.9050–0.9600)	0.26 (0.12–0.42)	0.9825 (0.9721–0.9891)	0.69 (0.55–0.79)

^a^ Daily survival rate.

^b^ Daily survival rate exponentiated to average Least Bittern (17 days) and Common Gallinule (21 days) incubation length.

We fit models of nest fate for combined years as well as for 2021 alone because several nest context variables collected during 2021 (i.e., nest visibility, habitat openness, and stem density) were not collected in 2020 (Tables S3 and S4). Least Bittern nest survival in 2021 increased with day of year (*w*_*i*_ = 0.40; *β*_day of year_ = 0.0812.; 85% CI: 0.0251 to 0.1372) and decreased with increasing visibility (*w*_*i*_ = 0.23; *β*_visibility_ = −1.2041; 85% CI: −2.0783 to −0.3299). Additionally, Common Gallinule nest survival in 2021 increased with emergent vegetation height (*w*_*i*_ = 0.28; *β*_emergent vegetation height_ = 0.0297; 85% CI: −0.0113 to 0.0482), water depth (*w*_*i*_ = 0.19; *β*_water depth_ = 0.0317; 85% CI: −0.0101 to 0.0533), and nest height (*w*_*i*_ = 0.18; *β*_nest height_ = 0.0168; 85% CI: 0.0058 to 0.0278) and decreased with increasing distance to shore (*w*_*i*_ = 0.19; *β*_distance to shore_ = −0.0021; 85% CI: −0.0041 to −0.0002) and later in the year (*w*_*i*_ = 0.27; *β*_day of year_ = −0.0458; 85% CI: −0.0777 to −0.0138), although the confidence intervals for both vegetation height and water depth included zero indicating weaker evidence for these relationships.

#### 3.3.3 Predator‐specific nest predation

**FIGURE 5:**
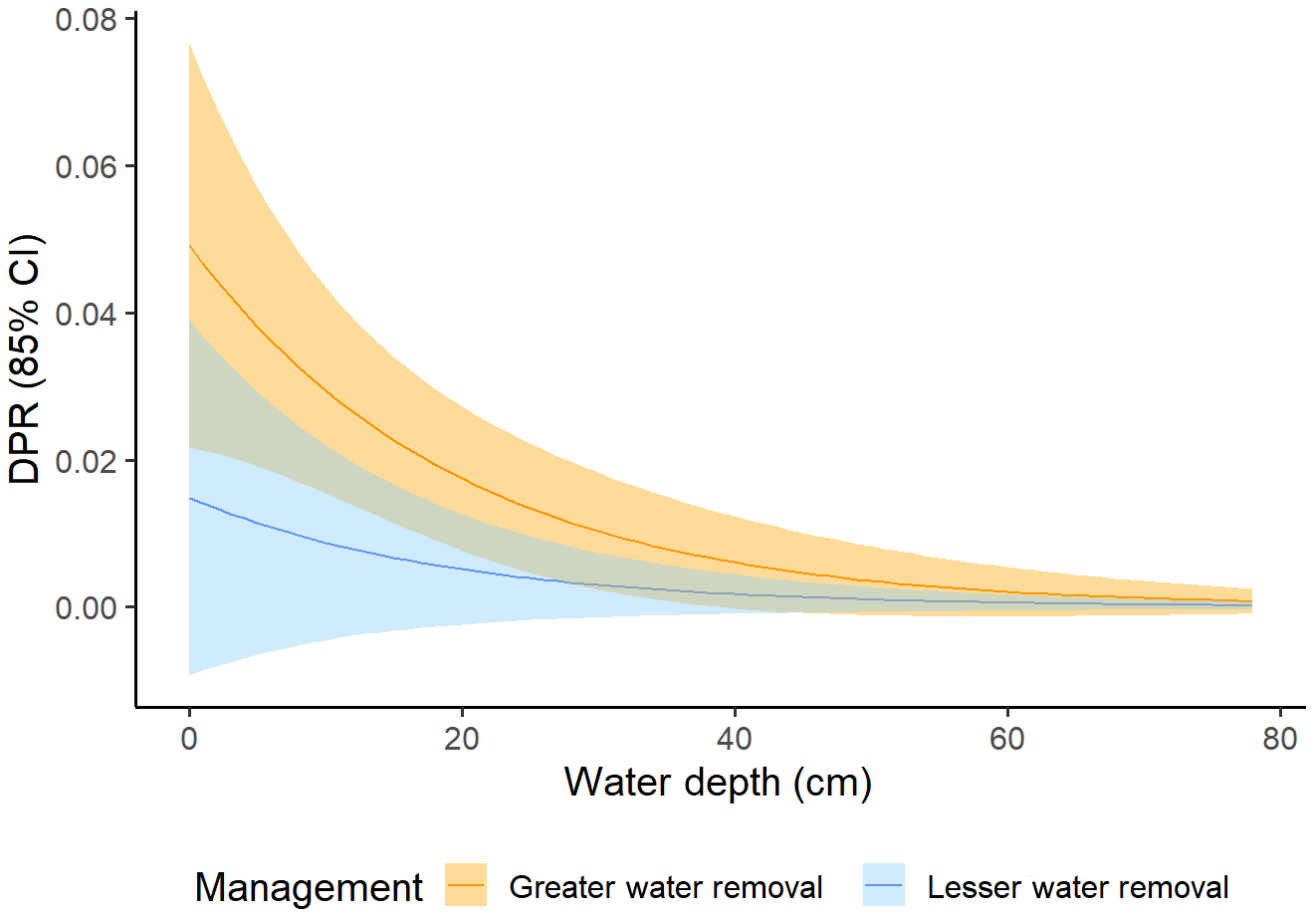
Daily predation rate (DPR) of all marsh bird nests with cameras (*n* = 78) by mammals in a year of high water management intensity (2020) and a year of low water management intensity (2021) at Emiquon Preserve, Illinois, USA, as a function of water depth (cm) and management intensity/year.

**FIGURE 6:**
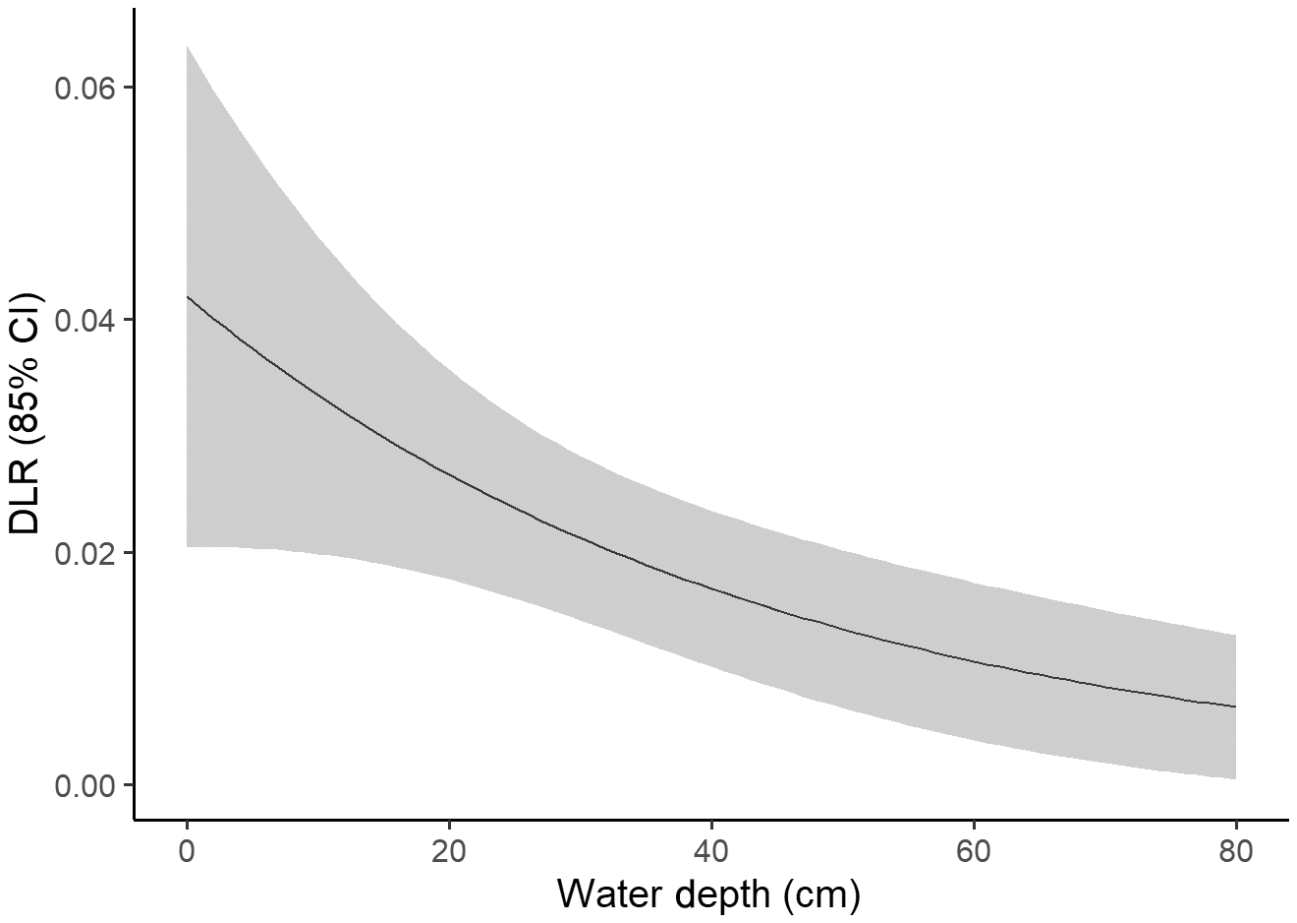
Daily loss rate (DLR) of all marsh bird nests with cameras (*n* = 78) by losses identified as other (unknown predators, abandonments) in 2020 and 2021 at Emiquon Preserve, Illinois, USA as a function of water depth (cm).

The best fit model contained the variables for water depth and year of greater/lesser water removal (*w*_*i*_ = 0.75) (Table S5). Daily nest loss rates at the mean nest site water depth (*x̄* = 35.2 cm) attributed to mammals, snakes, birds, and others in 2020 were 0.015, 0.013, 0.005, and 0.017, respectively, and 0.007, <0.001, 0.004, and 0.025 in 2021 (Table S6). Mammalian predation increased as water depth decreased (range: 0–32 cm; *x̄* = 11.8 cm; *β*_water depth_ = −0.0006; 85% CI: −0.0008 to −0.0003) and decreased from the year with rapid drawdown of water (2020) to the year with more stable conditions (2021) (*β*_year of greater/lesser water removal_ = −0.0177; 85% CI: −0.0277 to −0.0076) (Table 3, Figure 5). Alternatively, water depths of nests not visited by predators ranged from 0 to 83 cm (*x̄* = 35.5 cm). Nest failure due to other causes (i.e. abandonment and unknown losses) also increased in shallower water (range: 0–50 cm; *x̄* = 25.6 cm; *β*_water depth_ = −0.0006; 85% CI: −0.0010 to −0.0002) (Table 3, Figure 6). Predation by snakes only occurred in 2020, the year of greater water removal (*β*_year of greater/lesser water removal_ = −0.0112; 85% CI: −0.0183 to −0.0040) (Table 3). Daily predation rate for snakes in 2021 was estimated at <0.001 as we were unable to fix this value at zero.

**TABLE 3:** Parameter estimates for competitive predator‐specific multinomial models of nest failure at Emiquon Preserve, Illinois, USA in 2020 and 2021.

Variable	Predator class	Coefficient (*β*)	85% Confidence interval
Water depth	**Mammal**	**−0.0006**	**−0.0008 to −0.0003**
	Snake	0.0002	−0.0004 to 0
	Bird	0.0001	−0.0001 to 0.0003
	**Other**	**−0.0006**	**−0.0010 to −0.0002**
Year of greater/lesser water removal	**Mammal**	**−0.0177**	**−0.0277 to −0.0076**
	**Snake**	**−0.0112**	**−0.0183 to −0.0040**
	Bird	−0.0013	−0.0085 to 0.0059
	Other	0.0089	−0.0065 to 0.0243

Significant predator classes do not include zero in their confidence interval and are bolded.

## 4 DISCUSSION

Retaining deeper water below nests improves nest survival of vulnerable marsh birds, however, removal of water, especially at greater intensities, during peak nesting eliminates the protection afforded by water and may increase access to nests by mammalian predators. Active dewatering of emergent wetlands as a management tool during the growing season can be used to produce vegetation with energy‐rich seeds and tubers to feed migrating and wintering waterfowl, as well as create emergent vegetation for marsh birds, mudflats for shorebirds, support diverse populations of invertebrates, and facilitate habitat use by furbearers, such as racoons and minks, typically deterred by deeper water (Bradshaw et al., 2020; Fournier et al., 2019; Fredrickson & Taylor, 1982; Lane & Jensen, 1999; Weller & Spatcher, 1965).

Least Bitterns and Common Gallinules are known to select nest sites in dense inundated vegetation, avoiding dry areas altogether (Fournier et al., 2021), potentially due to greater predation risk in shallow water or dry locations, greater proximity to prey, or protection from weather (Holway, 1991; Moore et al., 2009; Weller, 1961). The negative impact of shore proximity on Least Bittern nest survival may ultimately result from nests being closer to areas where nest predators are more abundant or active (Báldi & Batáry, 2005; Batáry & Báldi, 2004; Jedlikowski et al., 2015). For both Least Bitterns and Common Gallinules, the deep‐water marsh interior may deter for mammalian predators (Hoover, 2005). Raccoons, mink, and fox snakes are often associated with areas adjacent to wetlands and are known to use wetland edges (Ahlers et al., 2016; Barding & Nelson, 2008; Weatherhead & Blouin‐Demers, 2004). Proximity of nests to edges may increase predation risk by increasing habitat overlap between predator and prey (Báldi & Batáry, 2005; Batáry & Báldi, 2004; Ellis et al., 2020). Similar to our results, the absence of water has been found to increase nest predation of Least Bitterns by connecting edge habitat to marsh interior (Post, 1998). We did not observe this relationship in Common Gallinules, presumably because Common Gallinules are associated with deeper water towards the marsh interior (Moore et al., 2009; Weller, 1961).

Deeper water during the nesting period has been observed to be an important predictor of predation risk by mammals specifically, raccoons (Barding & Nelson, 2008; Picman & Schriml, 1994). In particular, high water levels may reduce predation risk by deterring mammalian movement and decreasing search efficiency (Hoover, 2005; Jobin & Picman, 1997; Picman et al., 1993; Picman & Schriml, 1994). Water depth was also an important predictor for losses from predators we failed to identify because of camera malfunctions. We suspect many unidentified predators were likely mammals, as they more frequently knocked over nests during predation events.

Nest abandonment was also a problem in some circumstances, particularly for Common Gallinules. For this species, birds were more likely to abandon nests with cameras than those without, although it is unclear whether this is related to bird responses to the camera or something related to the nests that were more likely to receive cameras. Abandonments have been observed to be influenced by the placement of cameras at nests (Renfrew & Ribic, 2003). Further, nesting birds evaluate risks, which likely caused them to abandon nests in shallow water where they were at greater risk of predation by mammals or following an encounter with a predator (Lima, 2009; Weller, 1961).

We did not observe fox snakes depredating nests in 2021, likely because the lower starting water levels in 2021 led to greater distance between inundated wetland vegetation and adjacent non‐wetland habitats relative to 2020. Snakes are ectotherms and seek thermally hospitable and predator‐free areas, such as wetland edges (Blouin‐Demers & Weatherhead, 2001; Lee, 2006). Outside of the vegetated wetland edge and on exposed mudflat, a snake risks depredation (Weatherhead & Blouin‐Demers, 2004). In 2020, high water levels at the start of the breeding season created marsh habitat with an inundated and vegetated edge, ideal for both nesting marsh birds and snakes. However, in 2021, the edge vegetation was not inundated and amenable to nesting marsh birds, so the nearest nests were located nearer the marsh interior which was only accessible by passing through exposed mudflat and water.

In 2021, when habitat conditions were largely unchanging between nests due to the late season low intensity dewatering, our analysis including additional variables collected only in 2021 revealed a relationship between nest survival and factors affecting nest visibility and activity. More visible nests had poorer survival, and studies of open‐nesting birds in the tropics, desert, and grasslands, suggest when habitat conditions have facilitated access to or visibility of a nest, activity at or around a nest can act as a visual or olfactory cue to alert predators (Colombelli‐Négrel & Kleindorfer, 2005; Martin et al., 2000; Skutch, 1949). The day of year also impacted nest survival to varying degrees, with greater Least Bittern nest survival seen later in the season and greater Common Gallinule nest survival seen earlier in the season. This result was likely due to timing and availability of species nest site selection preferences (i.e., vegetation height and density, water depth; Fournier et al., 2021) and nesting periods overlapping or failing to overlap with favorable climatic conditions, food availability, or predator activity (Cain et al., 2010; Siikamäki, 1998; Sperry et al., 2008).

In conclusion, our results suggest retaining water in emergent wetlands during peak nesting deters mammalian predators and improves overall nest survival of secretive marsh birds and that identifying habitat components associated with nest predators may help to better manage nest loss. Although relatively stable water conditions appear to promote greater reproductive success by conservation‐priority wetland birds, management goals, such as moist‐soil seed production, invasive species control, and emergent wetland habitat creation, can likely still be met while protecting vulnerable nesting birds by managing water levels outside of active breeding periods (May–June) (Bradshaw et al., 2020; Jobin et al., 2009). More work, however, is needed to better understand the tradeoffs associated with retaining water for nesting marsh birds and effects of seed production for fall and spring migration of waterfowl. Dewatering later in the summer may shorten the growing period for emergent and moist soil plants, potentially leading to decreased subsequent habitat structure and food availability. The literature on the effects of drawdown timing on later habitat quality is inconclusive (Bellrose, 1941; Bowyer et al., 2005; Fleming et al., 2012; Fournier et al., 2019; Fredrickson & Taylor, 1982; Hine et al., 2017; Merendino et al., 1990) and local site context and other factors likely play important additional roles in influencing the impacts of drawdown timing on habitat use of waterbirds.

## AUTHOR CONTRIBUTIONS

**Stephanie M. Schmidt:** Data curation (lead); formal analysis (lead); funding acquisition (supporting); investigation (lead); methodology (lead); supervision (supporting); validation (supporting); visualization (lead); writing – original draft (lead); writing – review and editing (lead). **Auriel M. V. Fournier:** Conceptualization (lead); data curation (supporting); formal analysis (supporting); funding acquisition (lead); methodology (supporting); project administration (lead); resources (lead); software (lead); supervision (lead); validation (lead); visualization (supporting); writing – original draft (supporting); writing – review and editing (supporting). **Joshua M. Osborn:** Investigation (supporting); methodology (supporting); resources (lead); software (lead); visualization (lead); writing – review and editing (supporting). **Thomas J. Benson:** Conceptualization (lead); data curation (supporting); formal analysis (supporting); funding acquisition (lead); methodology (supporting); project administration (lead); resources (lead); software (lead); supervision (lead); validation (lead); visualization (supporting); writing – original draft (supporting); writing – review and editing (supporting).

## FUNDING INFORMATION

Funding was provided by a State Wildlife Grant (T‐122‐R) from the Illinois Department of Natural Resources and the United States Fish and Wildlife Service, as well as through the Illinois Ornithological Society (DuPage Birding Club, Chicago Audubon Society) and American Ornithological Society.

## CONFLICT OF INTEREST STATEMENT

The authors declare that they have no conflicts of interest.

## Supporting information


Table S1

## Data Availability

Data and code associated with our study are not yet publicly available, but can be accessed through this reviewer link: https://datadryad.org/stash/share/4bxI58_PcbZ‐NI4uBnidOlZ3v3YvHInXz78atZl83n0. Forthcoming on Dryad through this link: https://doi.org/10.5061/dryad.0zpc8673j.
