# Supplementary material for: Water depth influences survival and predator‐specific patterns of nest loss in three secretive marsh bird species
Source: Ecol Evol. 2023 Dec 11;13(12):e10823. doi: 10.1002/ece3.10823 (PMC10714062; doi:10.1002/ece3.10823)
Supplement: Supplementary file 1 — Table S1 [file ECE3-13-e10823-s001.docx]

**SUPPLEMENTARY MATERIALS**

Table S1. Model selection results for logistic exposure models explaining Least Bittern nest survival in 2020 and 2021at Emiquon Preserve, Illinois, USA as a function of nest context and temporal/biological variables.

| Nest Context Models 2020 and 2021 | | | | |
| --- | --- | --- | --- | --- |
| Model | *k* | AIC_c_ | Δ AIC_c_ | *w_i_* |
| Water Depth + Distance to Shore + Year of Greater/Lesser Water Removal | 4 | 241.39 | 0.00 | 0.84 |
| Water Depth + Distance to Shore | 3 | 245.15 | 3.77 | 0.13 |
| Water Depth | 2 | 247.86 | 6.47 | 0.03 |
| Year of Greater/Lesser Water Removal | 2 | 256.75 | 15.36 | 0.00 |
| Distance to Shore | 2 | 263.64 | 22.25 | 0.00 |
| Constant Survival | 1 | 268.45 | 27.07 | 0.00 |
| Avg. Emergent Vegetation Height | 2 | 269.52 | 28.13 | 0.00 |
| Nest Height | 2 | 270.41 | 29.02 | 0.00 |
| Nest Height × Avg. Emergent Vegetation Height | 4 | 272.37 | 30.98 | 0.00 |
| Temporal/Biological Models and Competitive Nest Context Models 2020 and 2021 | | | | |
| Model | *k* | AIC_c_ | Δ AIC_c_ | *w_i_* |
| Water Depth + Distance to Shore + Year of Greater/Lesser Water Removal | 4 | 241.39 | 0.00 | 1.00 |
| Constant Survival | 1 | 268.45 | 27.07 | 0.00 |
| Clutch Size | 2 | 269.84 | 28.45 | 0.00 |
| Nest Stage | 2 | 270.19 | 28.80 | 0.00 |
| Day of Year | 2 | 270.44 | 29.05 | 0.00 |

Table S2. Model selection results for logistic exposure models explaining Common Gallinule nest survival in 2020 and 2021 at Emiquon Preserve, Illinois, USA as a function of nest context and temporal/biological variables.

| Nest Context Models 2020 and 2021 | | | | |
| --- | --- | --- | --- | --- |
| Model | *k* | AIC_c_ | Δ AIC_c_ | *w_i_* |
| Water Depth + Year of Greater/Lesser Water Removal | 3 | 194.93 | 0.00 | 0.80 |
| Water Depth | 2 | 199.68 | 4.75 | 0.07 |
| Nest Height × Avg. Emergent Vegetation Height | 4 | 200.22 | 5.29 | 0.06 |
| Water Depth + Distance to Shore | 3 | 201.17 | 6.24 | 0.04 |
| Year of Greater/Lesser Water Removal | 2 | 203.12 | 8.19 | 0.01 |
| Nest Height | 2 | 203.47 | 8.54 | 0.01 |
| Avg. Emergent Vegetation Height | 2 | 203.75 | 8.82 | 0.01 |
| Constant Survival | 1 | 210.47 | 15.54 | 0.00 |
| Distance to Shore | 2 | 212.49 | 17.56 | 0.00 |
| Temporal/Biological Models and Competitive Nest Context Models 2020 and 2021 | | | | |
| Model | *k* | AIC_c_ | Δ AIC_c_ | *w_i_* |
| Water Depth + Year of Greater/Lesser Water Removal | 3 | 194.93 | 0.00 | 0.95 |
| Day of Year | 2 | 200.99 | 6.06 | 0.05 |
| Constant Survival | 1 | 210.47 | 15.54 | 0.00 |
| Nest Stage | 2 | 210.60 | 15.67 | 0.00 |
| Clutch Size | 2 | 211.84 | 16.91 | 0.00 |

Table S3. Model selection results for logistic exposure models explaining Least Bittern nest survival in 2021 at Emiquon Preserve, Illinois, USA as a function of nest context and temporal/biological variables.

| Nest Context Models 2021 | | | | |
| --- | --- | --- | --- | --- |
| Model | *k* | AIC_c_ | Δ AIC_c_ | *w_i_* |
| Visibility | 2 | 66.76 | 0.00 | 0.28 |
| Habitat Openness + Visibility | 3 | 67.76 | 1.00 | 0.17 |
| Water Depth + Visibility | 3 | 68.07 | 1.31 | 0.14 |
| Constant Survival | 1 | 68.86 | 2.11 | 0.10 |
| Water Depth | 2 | 69.08 | 2.32 | 0.09 |
| Avg. Emergent Vegetation Height | 2 | 70.07 | 3.31 | 0.05 |
| Habitat Openness | 2 | 70.20 | 3.44 | 0.05 |
| Nest Height | 2 | 70.21 | 3.45 | 0.05 |
| Stem Density | 2 | 70.62 | 3.86 | 0.04 |
| Water Depth + Distance to Shore | 3 | 70.78 | 4.03 | 0.04 |
| Temporal/Biological Models and Top Ranked Nest Context Model 2021 | | | | |
| Model | *k* | AIC_c_ | Δ AIC_c_ | *w_i_* |
| Day of Year | 2 | 65.65 | 0.00 | 0.40 |
| Visibility | 2 | 66.76 | 1.10 | 0.23 |
| Nest Stage | 2 | 66.95 | 1.30 | 0.21 |
| Clutch Size | 2 | 68.83 | 3.18 | 0.08 |
| Constant Survival | 1 | 68.86 | 3.21 | 0.08 |

Table S4. Model selection results for logistic exposure models explaining Common Gallinule nest survival in 2021 at Emiquon Preserve, Illinois, USA as a function of nest context and temporal/biological variables.

| Nest Context Models 2021 | | | | |
| --- | --- | --- | --- | --- |
| Model | *k* | AIC_c_ | Δ AIC_c_ | AIC_c_ *w_i_* |
| Avg. Emergent Vegetation Height | 2 | 108.69 | 0.00 | 0.28 |
| Water Depth + Distance to Shore | 3 | 109.45 | 0.76 | 0.19 |
| Nest Height | 2 | 109.51 | 0.82 | 0.18 |
| Water Depth | 2 | 110.00 | 1.31 | 0.14 |
| Constant Survival | 1 | 111.88 | 3.19 | 0.06 |
| Water Depth + Visibility | 3 | 112.00 | 3.31 | 0.05 |
| Habitat Openness | 2 | 112.72 | 4.03 | 0.04 |
| Stem Density | 2 | 113.68 | 4.99 | 0.02 |
| Visibility | 2 | 113.91 | 5.22 | 0.02 |
| Habitat Openness + Visibility | 3 | 114.76 | 6.07 | 0.01 |
| Temporal/Biological Models and Top Ranked Nest Context Model 2021 | | | | |
| Model | *k* | AIC_c_ | Δ AIC_c_ | *w_i_* |
| Avg. Emergent Vegetation Height | 2 | 108.69 | 0.00 | 0.53 |
| Day of Year | 2 | 110.06 | 1.37 | 0.27 |
| Constant Survival | 1 | 111.88 | 3.19 | 0.11 |
| Nest Stage | 2 | 113.20 | 4.51 | 0.05 |
| Clutch Size | 2 | 113.91 | 5.22 | 0.04 |

Table S5. Model selection results for multinomial regressions explaining predator-specific patterns of nest failure in 2020 and 2021 at Emiquon Preserve, Illinois, USA as a function of nest context and temporal/biological variables.

| Nest Context Models 2020 and 2021 | | | | |
| --- | --- | --- | --- | --- |
| Model | *k* | AIC_c_ | Δ AIC_c_ | *w_i_* |
| Water Depth + Year of Greater/Lesser Water Removal | 12 | 404.13 | 0.00 | 0.75 |
| Water Depth | 8 | 406.57 | 2.44 | 0.22 |
| Year of Greater/Lesser Water Removal | 8 | 410.51 | 6.38 | 0.03 |
| Avg. Emergent Vegetation Height | 8 | 416.36 | 12.23 | 0.00 |
| Constant Survival | 4 | 416.65 | 12.52 | 0.00 |
| Nest Height | 8 | 421.12 | 16.99 | 0.00 |
| Distance to Shore | 8 | 423.03 | 18.90 | 0.00 |
| Nest Height × Avg. Emergent Vegetation Height | 16 | 428.27 | 24.14 | 0.00 |
| Temporal/Biological Models and Competitive Nest Context Models 2020 and 2021 | | | | |
| Model | *k* | AIC_c_ | Δ AIC_c_ | *w_i_* |
| Water Depth + Year of Greater/Lesser Water Removal | 12 | 404.13 | 0.00 | 0.74 |
| Water Depth | 8 | 406.57 | 2.44 | 0.22 |
| Year of Greater/Lesser Water Removal | 8 | 410.51 | 6.38 | 0.03 |
| Clutch Size | 8 | 412.99 | 8.86 | 0.01 |
| Day of Year | 8 | 414.12 | 9.99 | 0.00 |
| Constant Survival | 4 | 416.65 | 12.52 | 0.00 |
| Nest Stage | 8 | 420.36 | 16.23 | 0.00 |

Table S6. Predators identified at nests of marsh birds at Emiquon Preserve, Illinois, USA 2020-2021. Nests of marsh birds observed included Least Bittern (LEBI), and Common Gallinule (COGA).

| Predator Class | Predator Identity | Number of Events |
| --- | --- | --- |
| Mammal | Raccoon | 5 (3 COGA, 2 LEBI) |
|  | American Mink | 3 (3 LEBI) |
|  | Unidentified | 2 (2 LEBI) |
| Snake | Fox Snake | 5 (3 COGA, 2 LEBI) |
| Avian | Common Gallinule | 1 (1 COGA) |
|  | Marsh Wren | 2 (2 LEBI) |
|  | Brown-headed Cowbird | 1 (1 COGA) |
| Other | Unknown^a^ | 10 (4 COGA, 6 LEBI) |
|  | Abandoned^b^ | 9 (6 COGA, 3 LEBI) |
| Total |  | 38 |

^a^ Predators were unidentified on cameras due to cameras or nests coming down or camera battery failure.

^b^ Abandoned nests were included only if an incubator was confirmed, and a nest was abandoned if not attended for 24 hours.
